# The Brain (Cerebrum) as an Afferant or Incident Excitor to Nervous Periphery, and as Related to Volitional and Emotional Movements, Etc.

**Published:** 1875-01

**Authors:** E. F. Starr

**Affiliations:** Nacoochee, Ga.


### THE BRAIN (CEREBRUM) AS AN AFFERANT OR INCIDENT EXCITOR TO NERVOUS PERIPHERY, AND AS RELATED TO VOLITIONAL AND EMOTIONAL MOVEMENTS, Etc.

By E. F. STARR, M.D., of Nacoochee, Ga.

It is proposed in this article to reproduce, with some additions, the substance of an unpublished one written in the year 1852, previous to which time the writer had not seen the same views expressed elsewhere; but not having access to all the recent physiological works, he cannot say now how nearly some one may have given utterance to them, nor, on the other hand, how manifestly some clearer way may have been shown. The reader can judge.

The physiology of psychical movements has ever been involved in obscurity, and the true nature of such movements difficult of comprehension and explanation. They have been supposed by some to depend upon certain mysterious *soul* influences, acting in an unknown and inexplicable manner—they who thus believe not perceiving that the *soul* or psychical force during animal life can only act in accordance with certain laws in their relation to material substances. The exclamation,

> "And why the heart beats on, and how the brain
> Says to the foot, now move, now rest again,
> From age to age we search, and search in vain,"

may serve to indicate the depth of the mystery and the difficulty of the solution.

W. Tyler Smith in his physiological work on "Parturition and Obstetrics," says: "The anatomical mechanism by which emotion produces convulsion is not understood. An attempt has been made to call movements, resulting from impressions on the nerves of special sense, and the emotions they excite, reflex cerebral movements. But movements of this kind are clearly different altogether from the reflex physical movements; they may arise from the memory as well as from sensual impressions." * * * "We know that emotional movements may occur in limbs paralyed to voluntary movements. Therefore the mechanism of emotional movements and emotional convulsions can hardly be the same as the mechanism of voluntary motion. That the mechanism of puerpral convulsions is spinal, and not cerebral, is evident, because the fit may occur during apoplexy with perfect loss of voluntary motion. But it is still a great mystery how emotion and psychical and cerebral functions should affect the great physical organ of motor action. Here we have a chasm which we cannot bridge—at which the human intellect recoils—the separation between the physical and the psychical, soul and body, in the living economy." Despairing language this, yet only such as many are wont to use whose investigations fail to reach their mark; and such things as are hidden to *them* are counted as sealed mysteries, and further investigations deemed useless. But the human intellect, regardless of despairing individuals, marches steadily onward—slowly it may be, but surely nevertheless.

Emotions may be defined to be states or conditions of the mind produced by impressions made through the senses or sensory organs, or by the operations of the mind alone, when acting upon the recollections of former sensations or ideas. These states may be manifested in the various exhibitions of anger, fear, love, hatred, disgust, abhorrence, grief, joy, etc., and may move upon us so strongly as to drive us to perform various acts characteristic of the emotions they serve to represent. When these impressions are suddenly and violently made, or the susceptibility unusually great, severe physical effects may be produced, or even convulsions may ensue. In many cases of intense emotional states, especially in persons without mental or moral discipline, the will is subordinate, or yields a ready assent to the dictates of the emotions. On the other hand, in a different class of cases, it will rise superior to the circumstances, subdue the perturbation, and preserve due order.

The will is a quality or attribute of the mind, and seems in a sense to be the creature of motive ideas—acts at their suggestion—yet it has the power of calling up, restraining, combining and directing thoughts. Whether or not the mind is a distinct *entity*, and has its independent abode within the sphere of the cerebrum, is a question we do not propose to discuss; yet it would be contrary to well established facts of physiology to say that the mind or soul in any way whatever produces bodily action independently of a definite medium or mechanism. There is a mechanism upon which all these phenomena of motion depend, and if they are to be divested of their mystery and obscurity, it must doubtless be done by a proper appreciation and interpretation of this mechanism.

That our object may be clearly set forth, we will state here the following two propositions, viz:

1. The brain (cerebrum) sustains a relation to the whole voluntary muscular system similar to that which exists between an afferant or incident nerve and the muscle, or set of muscles with which it is reflexly connected, *i. e.*, the relation of incident periphery; and 2. The mind, in its various states, emotional and volitional, is the peculiar normal stimulus, excitant, or irritant of this great incident excitor organ, and the influence of the latter in producing motion is exerted through the medium of the spinal centre, as in ordinary reflex action; hence it follows that all motor actions may be arranged into two classes—physico-spinal-reflex and psychico-spinal-reflex.

In reference to the first of these propositions, it may be said that anatomical and physiological researches have shown that the brain is not a centre or true primum mobile of the motor system, but yet it has much to do with motion—it arouses the motor power and makes it active, and so does, to some extent, an irritated incident-excitor nerve in any other part of the body. It follows, then, as a quick logical conclusion, that it must be a periphery of that system, and, as such, must partake, in some degree, at least, of the nature of other peripheries of the same, but is possessed of peculiar endowments, from the nature of things, which render it susceptible to the impressions of very delicate agencies. The relations which subsist between it and the nervous and muscular systems are, from the necessity of things, general and intimate; and as it is the seat of thought and volition, it is needful that its connections be so arranged that it may control all the parts readily and with precision. The necessities of life demand this; and to this end there are the sensory tract and the motory tract—the one to convey and conduct the impressions which result in sensations from the different parts of the body to the brain; the other to convey incident impressions from the brain, in the shape of mandates of the will or emotional influences, to the spinal ganglia, thence to be reflected to the different parts of the body. From what has been said of its (the brain's) relation to and place in the motor system, it is inferable that the mechanism of its influence must be analogous, in a great degree, to that of other parts sustaining like relations, and that the medium or agency by, and the manner in, which it produces motion must be like to those by which other incident nerves excite it.

Let it not be understood that mine is "an attempt to call movements resulting from impressions on the nerves of special sense, and the emotions they excite, reflex cerebral movements;" for if I do not misinterpret, there are no such movements. The brain is not a mirror to reflect impressions, as is the maner of the spinal cord. It (or the mind) receives reports of impressions from the different parts of the body, and gives or issues orders for action, but does not reflect.

An irritation of the extremity of an incident nerve is followed by contraction in some particular muscle or muscles with which it is specially connected in reflex association; but when the brain is the seat of irritation, the effect may be elective or general, and its character will be determined by the character of the idea or state of the mind which impresses the brain. Incident nerves have ordinarily fixed relations to other reflex nerves of a limited number, and are not individually apt to produce movements of a general character, nor convulsions, unless the motor ganglia be first brought into a state of polarity or exalted excitability. The brain, however, may produce general movements by its more general control and power of impressing the centres, but the brain cannot act upon the muscular system save through the medium of the spinal cord; the latter, however, can and does influence the muscular system independently of the former. By this the medulla spinalis is made to appear wholly and soully the organ or centre of reflex locomotor function, and if so, the brain must exercise its influence in the manner of an incident excitor of the reflex functions of the centre; hence the similarity suggested in the first proposition becomes evident. But not only so—the volitional and emotional movements are effected through the agency of those efferent or reflex nerves which are known to act in connection and harmony with a class of incident excitors. From this it may be deduced that they (the afferant) act in a similar reflex capacity in relation to the impressions made upon the great psychical organ or cerebral periphery, and the similarity between volitional and emotional movements and ordinary reflex movements in this respect is marked. Then, also, if volitional and emotional movements are similar to the same, it is fair to conclude they are similar to each other—the difference being that one is guided by the co-ordinating will, and the other is not.

We come now to say of the second proposition, that the position that the mind in its various active states serves as an excitant or irritant to the cerebrum, as an incident organ, producing effects similar to those produced by the action of excitants upon ordinary incident nerves, derives support from sources and reflections, of which the following are a few: The ordinary spinal incident and reflex actions, extending from periphery to centre and from centre to periphery again, may be considered the type of all nervous motor functions; but to produce these actions it is necessary that an incitant be applied to the incident peripheries. The influence of an excitant is necessary, as well as a susceptible organ or medium, to the production of all motor actions; but mere movements only, without the guidance of an intelligent principle, could not serve any useful purpose or end in man; therefore, to place the locomotive apparatus under the influence and control of the thinking mind, it was necessary to have a development, organ or periphery which should be susceptible to the impressions of intellectual agencies, and capable of transmitting them to the general motor organ. So psychical movements depend upon the susceptibility of the brain to impressions produced by mental operations or conditions, and upon its faculty of conveying, as an incident excitor, reports of its impressions to the spinal ganglia, thence to be reflected to the muscles. In what particular manner the mind and the material brain act and react upon each other, in production and disintegration, as relative genetic cause and effect, we do not propose to inquire; we are only striving to evolve and simplify the philosophy of certain cerebral functions and of psychical movements.

That certain mental operations or states of the mind do produce spinal reflex actions may be shown by several illustrations; for instance, the engorgement of the male erectile organ by the contraction of certain muscles, confining the blood in its vessels, is an action of this reflex character, and may be brought about by the indulgence of voluptuous ideas. I am acquainted with an individual in whom the act of sneezing is excited by a similar state of the mind and the near approach and contact of the object promising indulgence of the aphrodisiac sense. The flushed countenance, hurried breathing and general physical perturbation excited by an angry state of the mind, are to be classed with the reflex motor manifestations. Irrepressible laughter, excited by funny ideas, is clearly of the same nature, or mento-spinal reflex; and the same may be said of involuntary sobbing and outcrying in consequence of intensely sorrowful ideas. These and other like instances show that spinal reflex movements result from states of the mind acting as excitors to the cerebral periphery. The movements resulting from these emotional states are mento-spinal-reflex, as are the volitional movements, and executed by the same mechanism, as it would not be consistent with the recognized rules of economy, in nature's works, to have two mechanisms for operations so similar.

In special reference to emotional movements, which have given rise to so much speculation, it is believed there need not be more difficulty in their explanation by this method of interpretation than there is in that of ordinary reflex phenomena; for, certain states of the mind, without any active interference of the will, or any volitional expenditure, effort, or command, may so impress the incident cerebral periphery that it may, in advance of the mandate of the will, so to speak, communicate the impression to the motor centres, or chain of spinal ganglia, and thus produce impulsive movements, or, in cases of idiosyncrasy or peculiar susceptibility, even convulsions. The will, let it be remembered, is not the only faculty or quality of the mind capable of affecting the brain; therefore, may not physical movements be caused by processes of cerebration whilst the will is incognito, or not sprung to action by its motive ideas? In various processes of intellectuality, may not the state of the cerebral periphery induced thereby call into action movements consonant and in harmony with the train of thought which may prevail at the time, when the will, if concerned at all, has given but the initiatory impulse? This is at least consistent with the idea that the brain is, in its essential nature, an incident periphery, and subject to mental influences. And are not the volitional movements, being congruous, executed because the state of the mind known as volitional impresses the brain as an incident excitor? and are not the incongruous emotional movements in like manner executed, because the state of the mind known as emotional impresses the brain in a similar relation? and are not both accomplished by the same mechanism? If the will be strong and well disciplined, it may check, restrain, control the emotional, by setting to work counter-actions, or restraining forces, or by producing impressions dissimilar to and stronger than the others; but if it be weak and uncultivated, it may be overpowered and subordinated by a crowd of strong emotions, and then physical and psychical movements may be blended in a vortex of incongruous manifestations.

We have spoken of the cerebrum as a nervous periphery of peculiar susceptibility, capable of being impressed by an æthereal agent, and of transmitting its impressions as an incident excitor to other parts, and of its local coincidence or co-existence with the mental faculties, etc.; but it has a still more peculiar relation to the nervous system than this. Upon a careful consideration of its functions, the writer is convinced that it is a double or two-fold periphery, a periphery between two centres, upon the reflex side of one and the incident side of the other; for this sensory ganglia, with their peripheries, and the motory ganglia, with their peripheries, including the cerebrum, seem clearly to constitute a compound organism with double reflex functions, or of a two-fold reflex character—a sensory reflex and a motory reflex—each having its own centre. An impression made on a nerve of special sense, as well as that made on one of the common sense of touch, is conveyed to the sensory centre or ganglia, and thence reflected to the cerebrum (just as an impression carried to the motory ganglia is reflected to the peripheral muscles), where ideal or intelligent cognizance is taken of it; and if it is of such a character as to require physical movements for the pleasure or preservation of the being, then orders, mandates, or mental influences, as the case may be, are issued to the motor department for such movements as may be necessary; while impressions made upon purely mortory nerves or fibres are conveyed to the motory ganglia, thence to be reflected to the muscles for such movements as have not their source in psychical influences.

It is believed that the nerves of special sense, with those of the common sense, have their own separate centre of action, because of both functional peculiarities and anatomic arrangement. Their efficiency depends altogether upon a state of consciousness, and consciousness or ability to perceive is a faculty of the mind, co-existent with a certain degree of integrity of the brain. Without consciousness there is no special sense nor any sensory activity, nor without consciousness can any irritation of the nerves of special sense produce or result in physical movements; but consciousness is not absolutely necessary to the activity of the functions of the motory system. The irritation of an incident nerve of the mortory system may produce physical movements in the absence of consciousness, but the same cannot be said of the incident nerves of the sensory system. Hence it may be concluded that the two classes of functions are sufficiently distinct to require two centres or mechanisms for their performance.

In conclusion, it may be remarked that there appears to be a similarity of characteristics existing between the nerves of special sense and the cerebrum, in the way of functional qualities or susceptibility to impressions, which consists briefly in this, that they are both insensible to ordinary impressions, not having the common sense of touch, and that they are alike impressible by imponderable and intangible agencies. Odors for the nostrils, sound for the ears, light for the eyes, and mind for the brain—neither one of which can be so applied as to impress the sentient extremities of the ordinary incident nerves; yet each respectively, decidedly and sufficiently impresses the peculiar organism to which it is specially adapted.

